# Supplementary material for: High-throughput screening of human genetic variants by pooled prime editing
Source: Cell Genom. 2025 Mar 21;5(4):100814. doi: 10.1016/j.xgen.2025.100814 (PMC12008803; doi:10.1016/j.xgen.2025.100814)
Supplement: Document S1. Figures S1–S14 [file mmc1.pdf]

Cell Genomics, Volume *5*

Supplemental information

High-throughput screening of human genetic variants by pooled prime editing

Michael Herger, Christina M. Kajba, Megan Buckley, Ana Cunha, Molly Strom, and Gregory M. Findlay

Figure S1

A

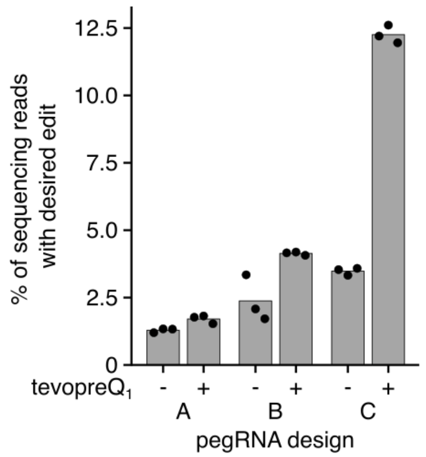


```
A: GCAAACATTCCACTACCACT...GACAtTCCCCAGa-GGTAGTGGAATG
B: GCAAACATTCCACTACCACT...GGTGACAtTCCCCAGT-GGTAGTGGAATGT
C: GCCCAAGTCAATGCAGAGGA...GGGAaTGTCACtATCC-TCTGCATTGACTT
```

B

```
original: GTTTTAGAGCTA-----GAAA-----TAGCAAGTTAAAATAAGGCTAGTCCGTTATCAACTT----GAAA----AAGTGGCACCGAGTCGGTGC
F+E:      GTTTAAGAGCTATGCTGGAAACAGCATAGCAAGTTTAAATAAGGCTAGTCCGTTATCAACTT----GAAA----AAGTGGCACCGAGTCGGTGC
F+E v1:   GTTTCAGAGCTATGCTGGAAACAGCATAGCAAGTTGAAATAAGGCTAGTCCGTTATCAACTT----GAAA----AAGTGGCACCGAGTCGGTGC
F+E v2:   GTTTCAGAGCTATGCTGGAAACAGCATAGCAAGTTGAAATAAGGCTAGTCCGTTATCAACTTGGACTTCGGTCCAAGTGGCACCGAGTCGGTGC
```

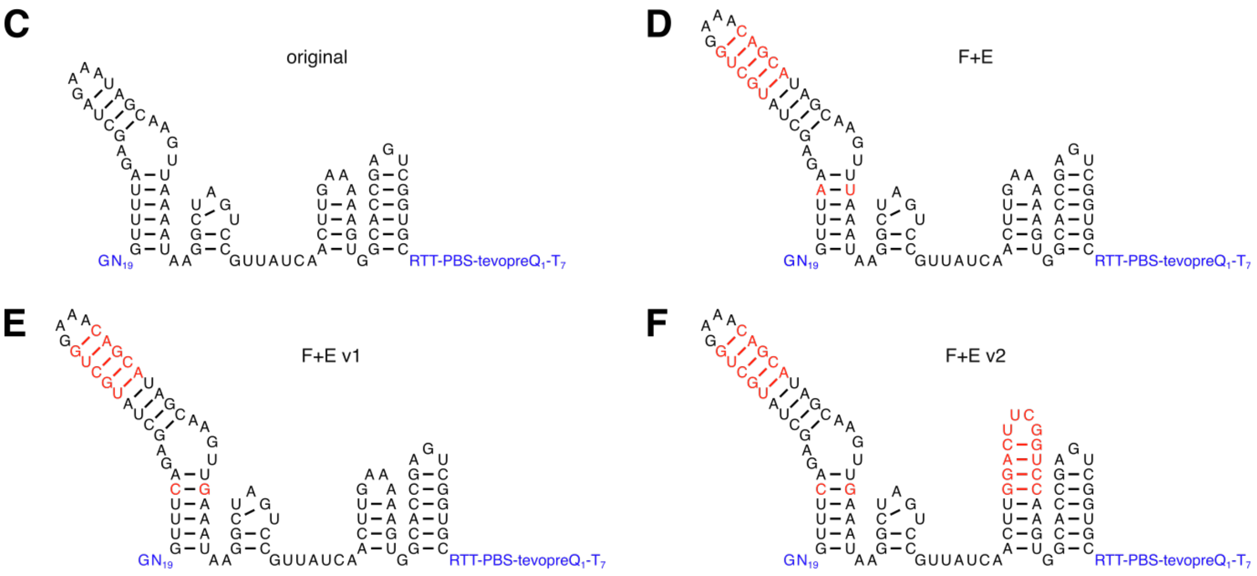

Figure S1. pegRNA optimization for *ATP1A1*-T804N edit in HEK293T and pegRNA scaffold designs, related to Figure 1.

(**A**) Comparison of PE efficiencies in HEK293T cells for installation of the T804N edit in *ATP1A1* using three pegRNA designs with and without the tevopreQ_1 motif. Values correspond to percentages of correct editing 4 days after transfection of pegRNA and PEmax-MLH1dn plasmids as determined by CRISPResso2[1] analysis of NGS reads. Individual transfection replicates are shown as dots. Below are sequences of pegRNA designs, labelled A-C, in the format "spacer…RTT-PBS", with programmed variants highlighted in red.

(**B**) Sequence alignment of pegRNA scaffolds used in this study for optimizing PE efficiency. Base changes against the original scaffold are colored red.

(**C-F**) The predicted secondary structure of each scaffold is shown. pegRNA-specific elements are colored blue.

Figure S2

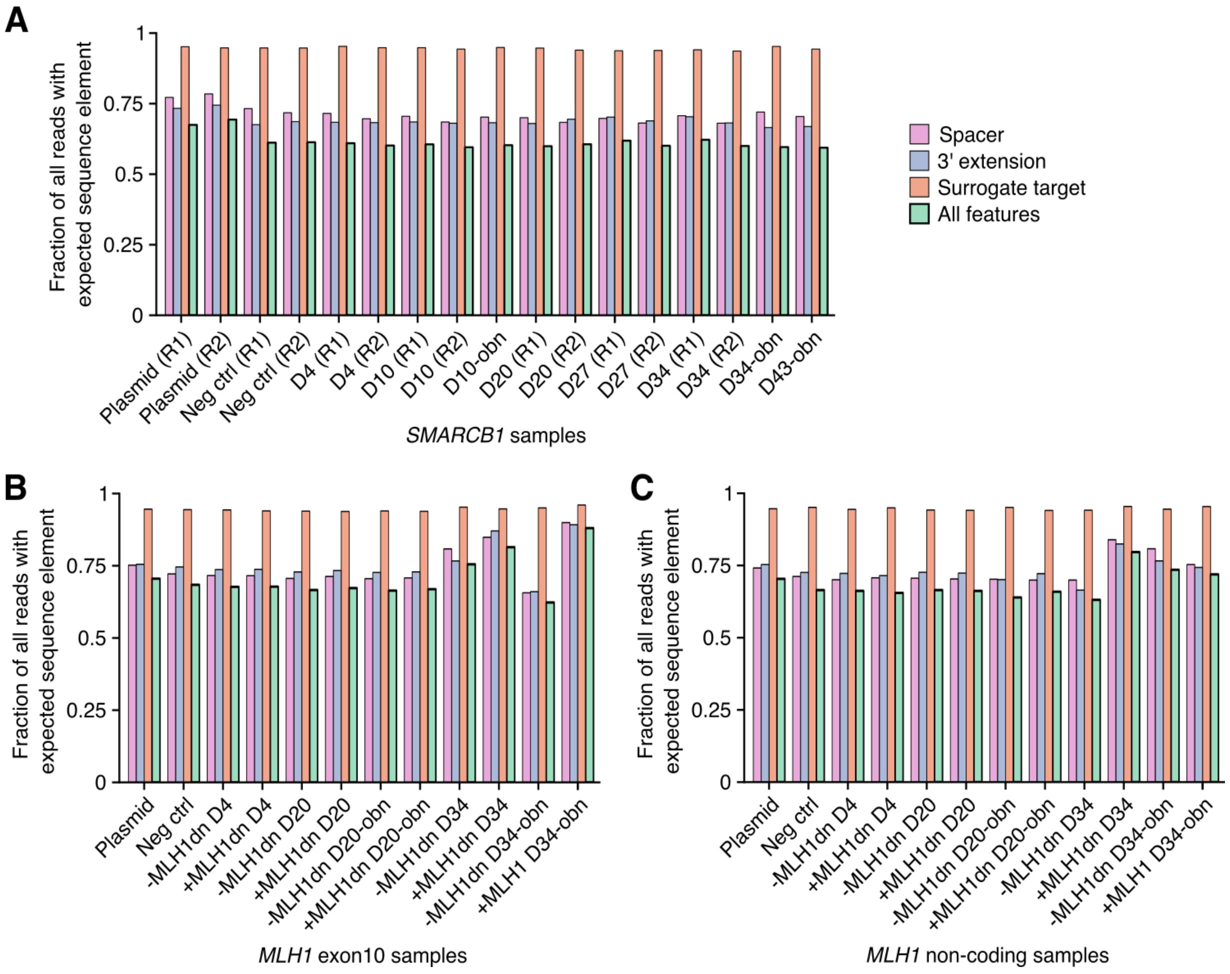

Figure S2. NGS read pre-processing of pegRNA-ST cassettes across experiments, related to Figure 1 and STAR Methods.

Results from pre-processing pegRNA-ST cassette sequencing reads for each sample across experiments: *SMARCB1* (**A**), *MLH1* exon 10 (**B**), and *MLH1* non-coding (**C**). Values correspond to the fraction of reads containing each correct sequence element (spacer, 3' extension, surrogate target or all elements) expected in the pegRNA construct, as identified by the read's pegRNA-specific barcode. Spacer and surrogate target sequences were allowed to differ by up to two base substitutions from the expected sequence to account for sequencing errors.

Figure S3

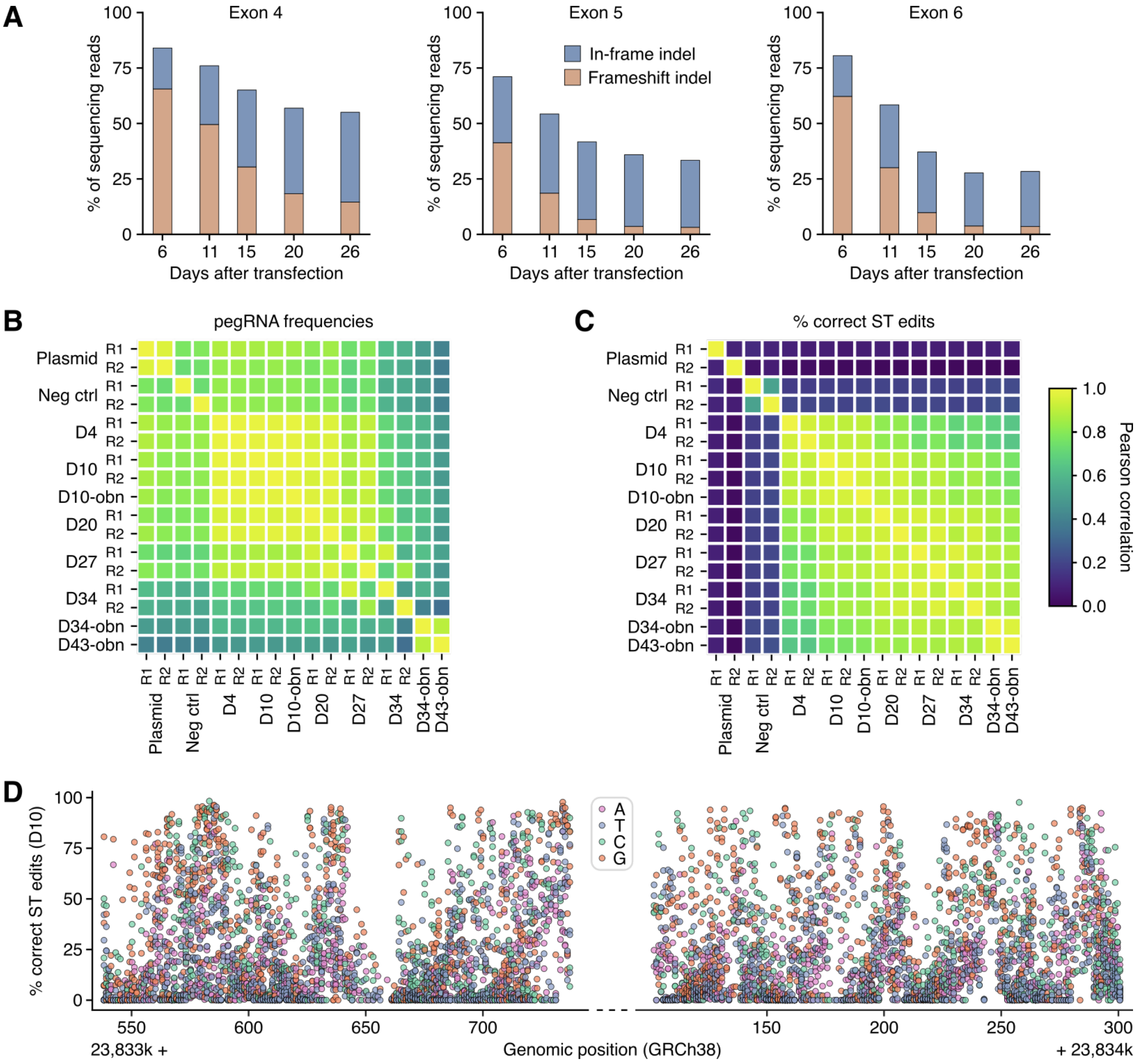

Figure S3. *SMARCB1* essentiality and pooled variant screening in HAP1, related to Figure 2.
(**A**) A time course was performed to assess in-frame and frameshifting indel percentages in exons 4, 5, and 6 of *SMARCB1* after Cas9-mediated editing in HAP1 cells. Indel rates were quantified from NGS reads using CRISPResso2.
(**B** and **C**) Heatmap of pairwise Pearson correlation coefficients between pegRNA frequencies (**B**) and correct ST editing percentages (**C**) across all collected samples of the *SMARCB1* variant screen, including the pegRNA plasmid pool (Plasmid) and transduced HAP1 wildtype cells (Neg ctrl).
(**D**) Scatter plot of correct ST editing percentages at 10 days post-transduction for each pegRNA (n = 6,902) targeting *SMARCB1* regions, colored by nucleotide substitution.

Figure S4

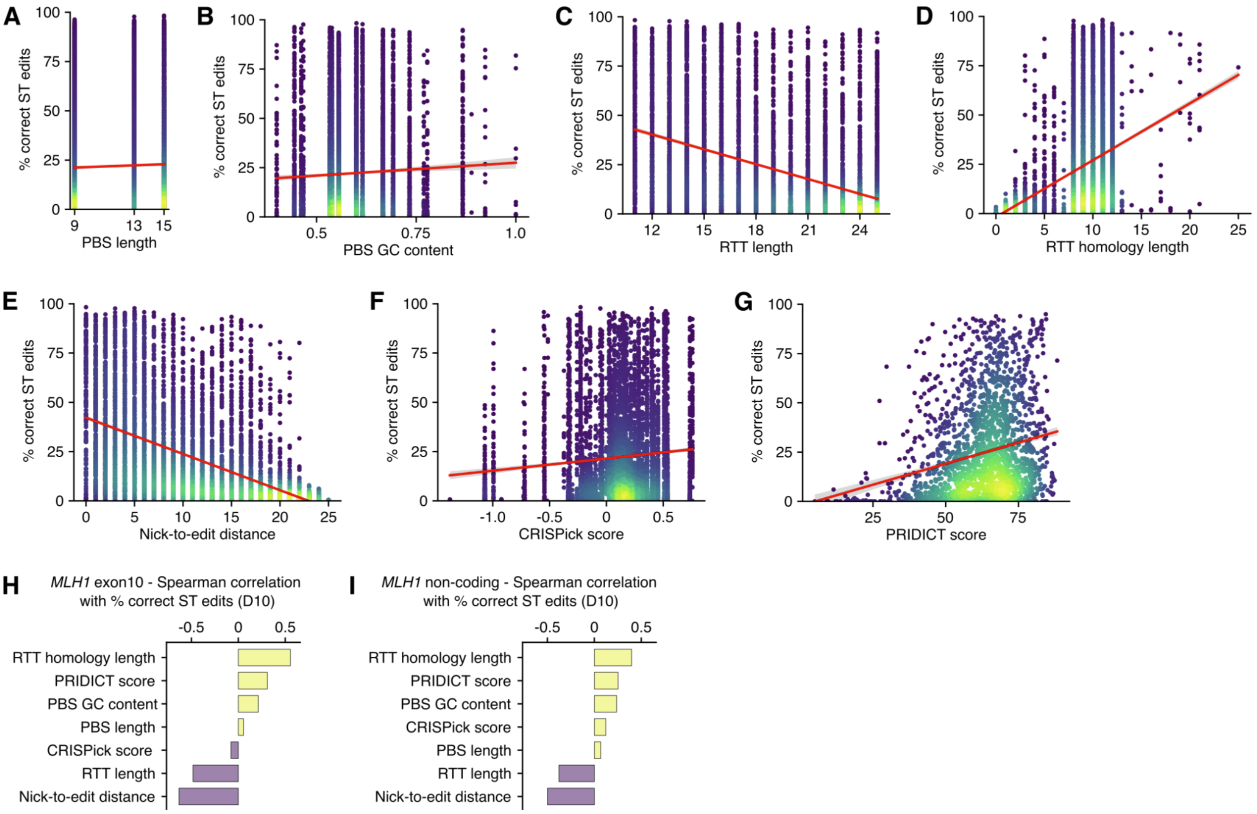

Figure S4. Impact of pegRNA features on correct ST editing, related to Figure 2.

(**A-G**) Percentage correct ST edits at day 10 averaged across the two non-ouabain treated experiments for *n* = 8,612 *SMARCB1*-targeting pegRNAs stratified by PBS length (**A**), PBS GC content (**B**), RTT length (**C**), (distal) RTT homology length (**D**), nick-to-edit distance (**E**), CRISPick score[2,3] (**F**), and PRIDICT score[4] (**G**). Points are colored by Gaussian kernel density estimation and a linear regression model (red line) with 95% confidence interval band (gray) is shown.

(**H and I**) Spearman correlations of percentage of correct ST edits at day 10 with different pegRNA features for *n* = 2,695 and *n* = 3,747 pegRNAs in the *MLH1* exon10 (**H**) and *MLH1* non-coding libraries (**I**), respectively.

Figure S5

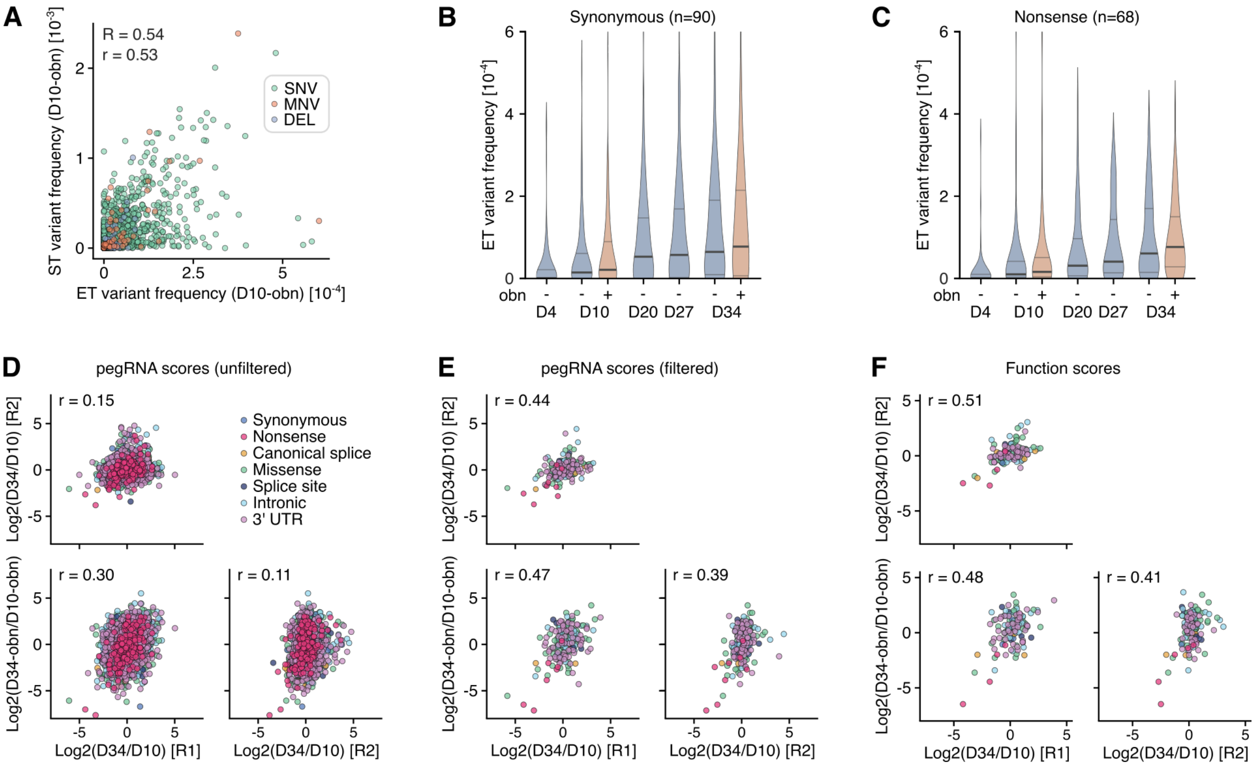

Figure S5. ST and ET variant frequency and score correlations for the *SMARCB1* screen, related to Figure 2.

(**A**) The correlation between ST editing and ET editing for each variant is plotted. Variants are colored by edit type and Spearman (R) and Pearson (r) correlation coefficients are shown.

(**B** and **C**) Variant frequencies at ETs across samples are plotted for all synonymous (**B**) and nonsense (**C**) variants. Frequencies were background-corrected using NGS data from negative control samples. Similar increases in frequency are observed over time for both synonymous and nonsense variants.

(**D**-**F**) Correlation plots of unfiltered (**D**) and filtered (**E**) pegRNA scores and function scores (**F**) for the *SMARCB1* variant screen between experiments with and without ouabain co-selection. Scores were calculated for pegRNA depletion between D10 and D34. pegRNAs passing filters were those with frequencies greater than $6{\times}10^{-5}$ in D10 samples and correct ST editing percentages greater than 75%. Pearson correlation coefficients (r) are shown for each pairwise comparison.

Figure S6

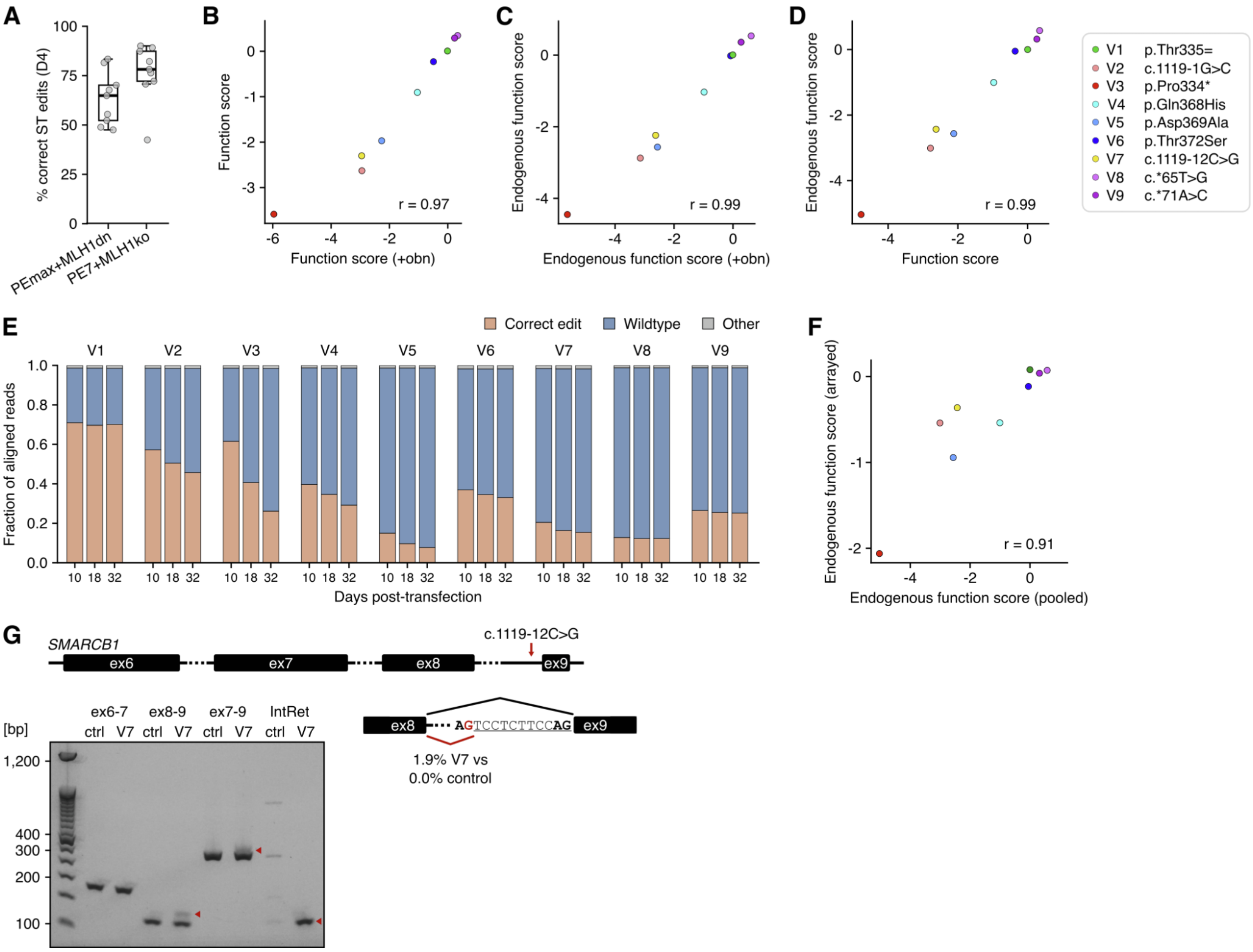

Figure S6. *SMARCB1* LoF variant validation, related to Figure 2.

(**A**) Comparison of ST editing efficiencies previously determined in HAP1:PEmax+MLH1dn and newly generated HAP1:PE7+MLH1KO cell lines 4 days after transduction with a pool of 9 pegRNAs used for variant effect validation. (Boxplot: bold line, median; boxes, IQR; whiskers to points within 1.5x IQR.)

(**B** and **C**) Function scores for the minipool experiment were calculated as log2-ratios of pegRNA frequencies (**B**) or ET variant frequencies (**C**) at day 32 over day 10, normalized to the score of a synonymous variant (p.Thr335= / “V1”) scored neutrally in the screen. Function scores are strongly correlated between conditions with (+obn) and without co-selection.

(**D**) Function scores derived from pegRNA frequencies and ET variant frequencies (averaged across conditions) are strongly correlated.

(**E**) Fraction of aligned sequencing reads corresponding to correct edits, unedited reference sequence (“wildtype”), and other editing outcomes as determined by CRISPResso2 analysis for samples to which individual pegRNAs were introduced. Data are shown for 3 timepoints.

(**F**) Endogenous function scores determined by testing pegRNAs individually (i.e., “arrayed”) are well-correlated to endogenous function scores from minipool screening (i.e., “pooled”).

(**G**) A potential splicing defect caused by c.1119-12C>G (“V7”) was evaluated with RT-qPCR, gel electrophoresis and NGS using cells to which either the V1 (control) or V7 pegRNA was introduced individually. Bands corresponding to RT-qPCR products across exon junctions 6-7, 8-9 and 7-9 are shown on the gel. An additional primer set was used to selectively amplify an 11-bp product of intron retention (“IntRet”) predicted to be caused by V7. Red arrows indicate observed differences between V1 and V7 consistent with aberrant splicing caused by V7. NGS analysis confirmed the presence of the IntRet isoform at 1.9% for V7 compared to 0.0% for V1 (averaged across n = 2 technical replicates).

Figure S7

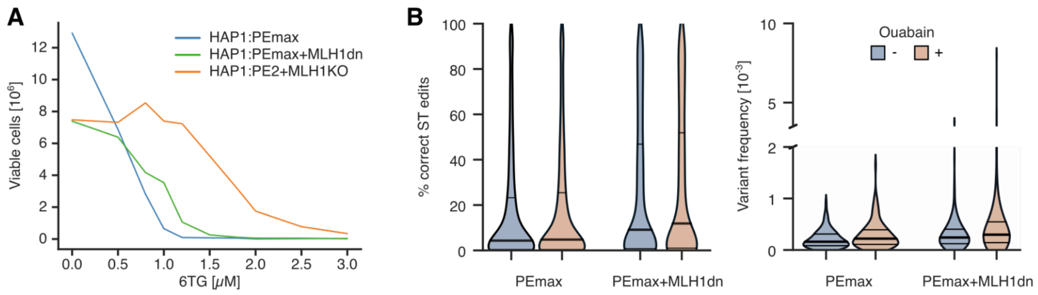

Figure S7. Effects of 6TG on growth and editing rates compared across HAP1 lines with modified MMR function, related to Figure 3.

(**A**) Viable cell counts are plotted by 6TG dose following 6 days treatment for HAP1:PEmax, HAP1:PEmax+MLH1dn, and HAP1:PE2+MLH1KO.

(**B**) Distributions of correct ST editing percentages for pegRNAs observed at frequencies greater than 1.4×10^{-4} in D20 samples (left), and distributions of ET variant frequencies in D20 samples (right) are shown for each condition. ET variants for which the log2-ratio of D20 frequency over D4 frequency was below 1.0 were excluded to limit potential impacts of sequencing error.

Figure S8

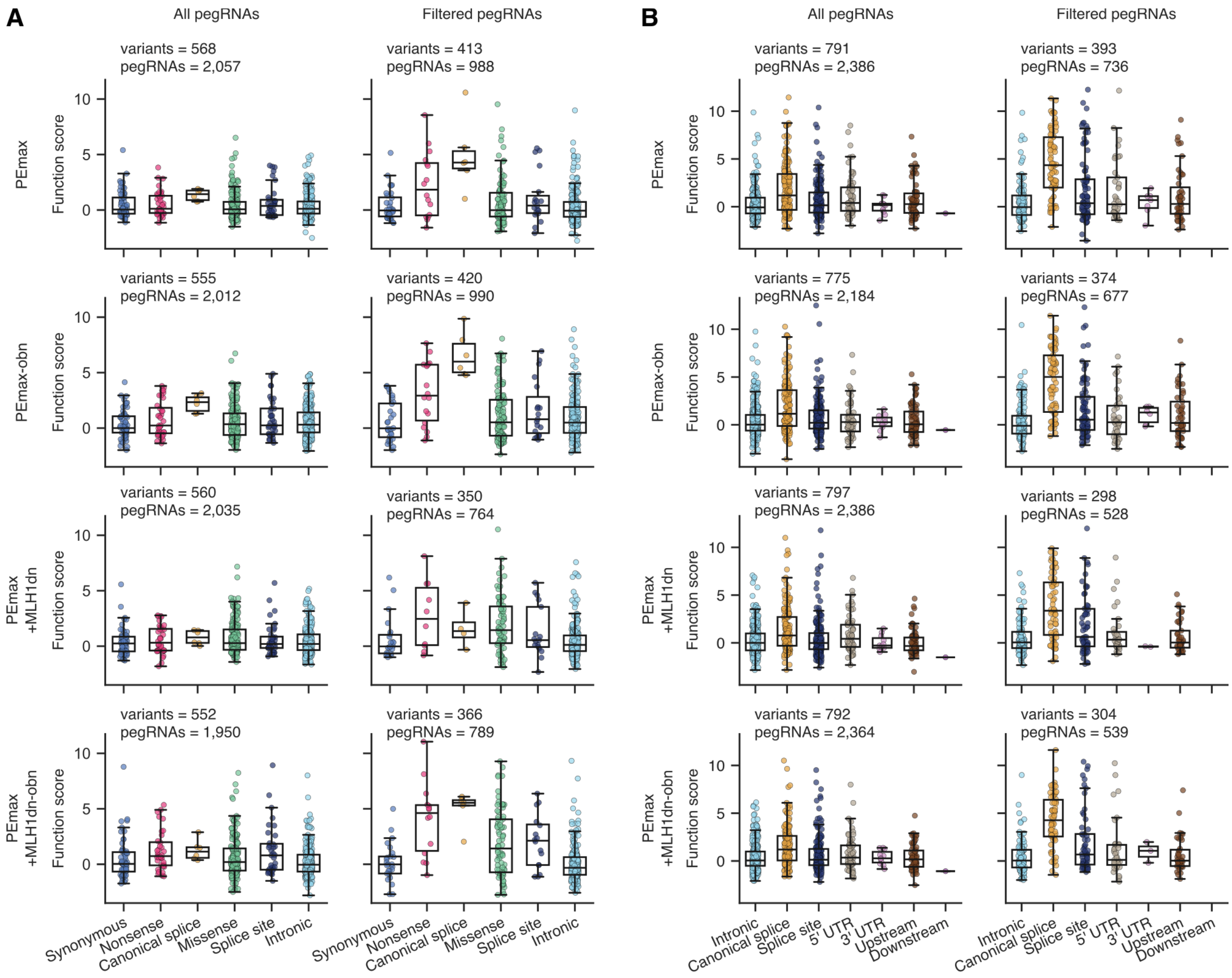

Figure S8. Filtering pegRNAs based on ST editing enables identification of LoF variants, related to Figures 3 and 4.

For each *MLH1* experiment, function scores were calculated for each variant assayed, using either all pegRNAs observed above a frequency threshold, or only the subset of those with correct ST editing percentages above a set threshold. Function scores, grouped by variant consequence, are shown before and after pegRNA filtering for the *MLH1* exon 10 screen (**A**) and the *MLH1* non-coding screen (**B**). ST editing thresholds to filter pegRNAs were set to 5% for HAP1:PEmax cells and 25% for HAP1:PEmax+MLH1dn cells.

Figure S9

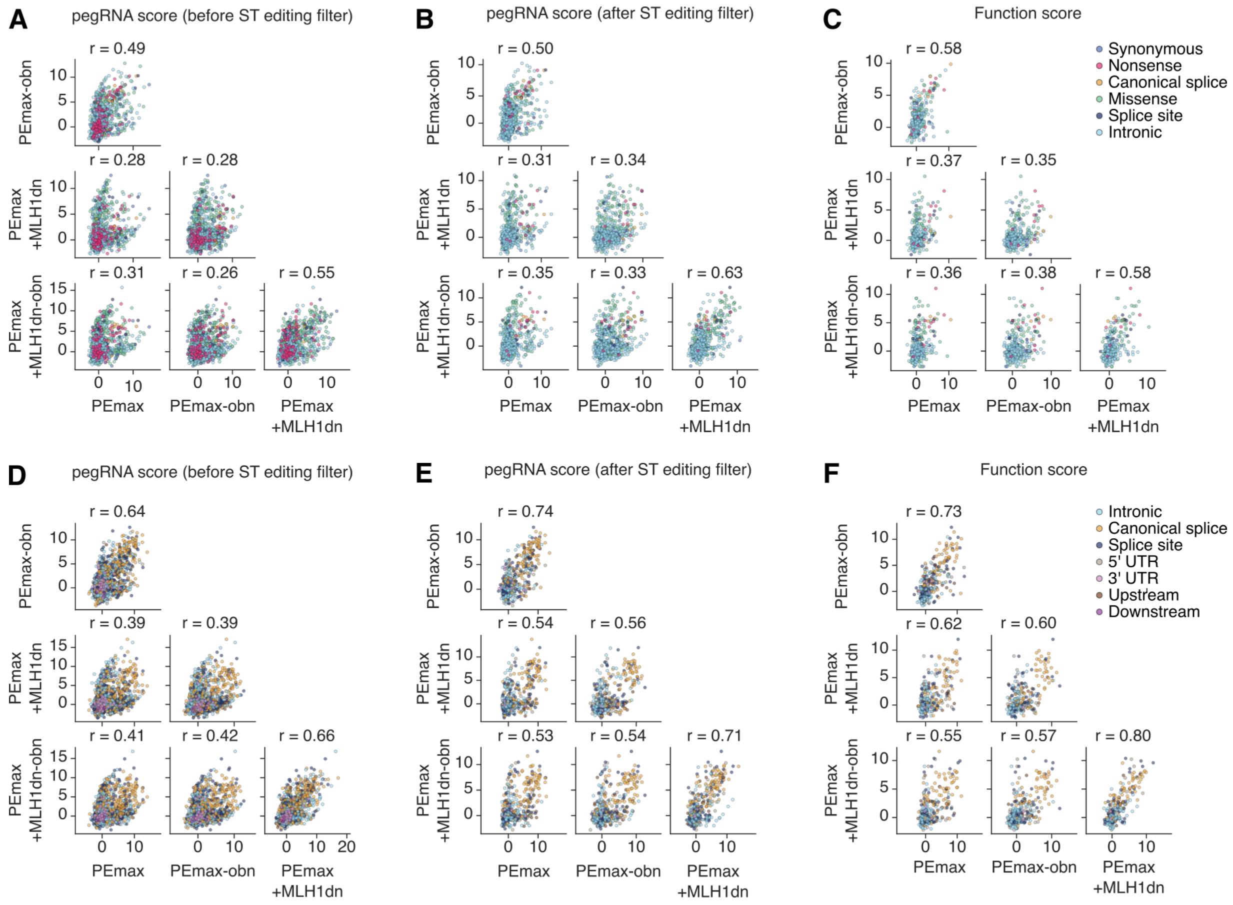

Figure S9. pegRNA score and function score reproducibility across experiments, related to Figures 3 and 4.
Correlations of unfiltered and filtered pegRNA scores and function scores across conditions for the *MLH1* exon 10 screen (**A**-**C**) and the non-coding screen (**D**-**F**) are shown. Correct ST editing thresholds were set to 5% for HAP1:PEmax cells and 25% for HAP1:PEmax+MLH1dn cells to remove inactive pegRNAs and produce function scores from the filtered set. Pearson correlation coefficients (r) are shown for each comparison.

Figure S10

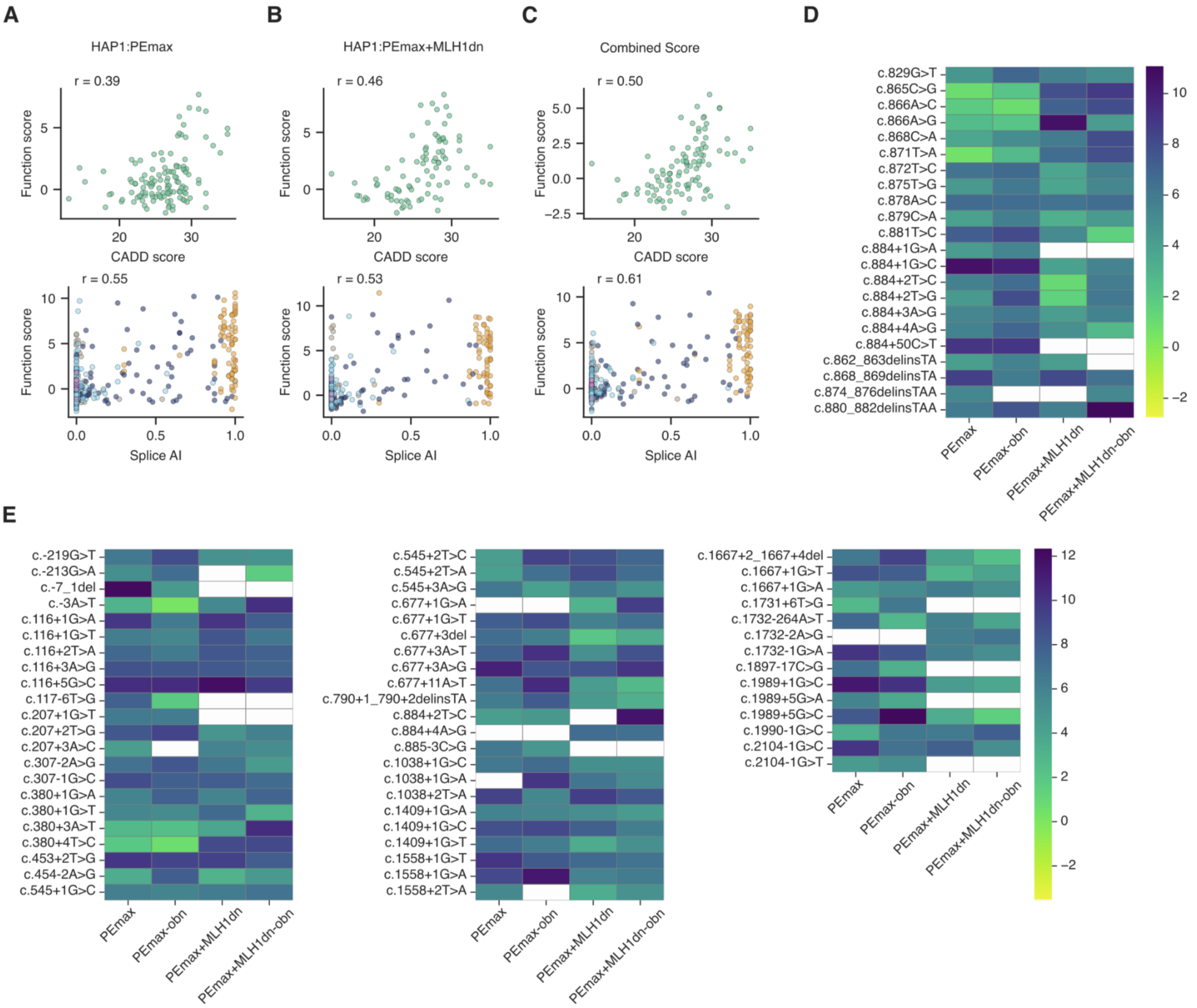

Figure S10. Consistency of *MLH1* variant effects between HAP1:PEmax and HAP1:PEmax+MLH1dn, related to Figures 3 and 4.

(**A-C**) Correlation between function scores and CADD scores (top), and between function scores and SpliceAI scores (bottom) using data generated in HAP1:PEmax only (**A**), in HAP1:PEmax+MLH1dn only (**B**), or by combining data from both HAP1:PEmax and HAP1:PEmax+MLH1dn (**C**). Pearson correlation coefficients (r) are shown for each comparison. (**D-E**) Heatmaps of function scores across the four experimental conditions for all variants deemed LoF in the exon 10 screen (**D**) and for all variants deemed LoF in the non-coding screen (**E**) are shown. The color scale corresponds to the minimum and maximum score observed in any condition.

Figure S11

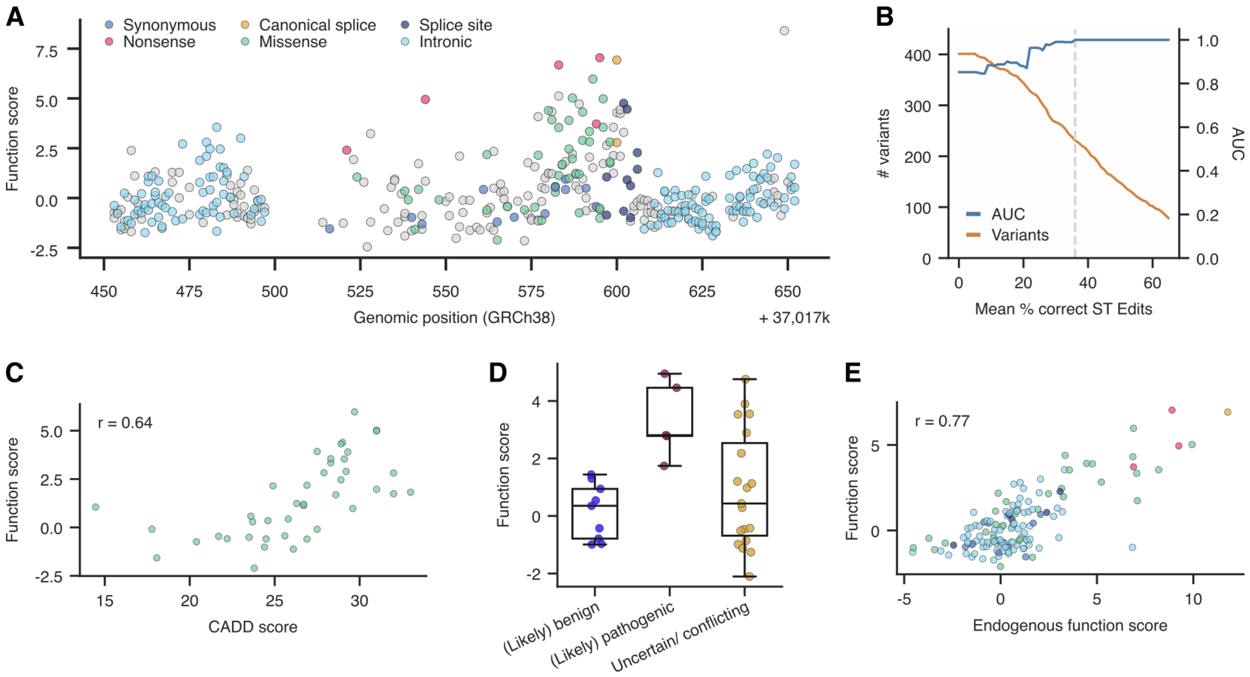

Figure S11. More stringent filtering of pegRNAs further improves data quality for variants assayed in *MLH1* exon 10, related to Figure 3.

(**A**) Function scores for n = 231 variants scored using an average correct ST editing threshold of 36% are plotted by position. (Gray dots indicate variants scored in Figure 3C with ST editing percentages below 36%.)

(**B**) AUC values for distinguishing LoF variants (blue) are plotted as a function of the mean ST editing threshold applied. For this analysis, synonymous variants were defined as neutral and nonsense and canonical splice variants as LoF. The orange line indicates the number of variants retained at each threshold, and the dashed line indicates the high-stringency threshold of 36%, above which AUC = 1.0.

(**C**) The correlation between function scores and CADD scores is plotted for n = 42 missense variants passing the high-stringency threshold.

(**D**) The boxplot shows function scores for n = 33 variants passing the high-stringency threshold by ClinVar pathogenicity status (bold line, median; boxes, IQR; whiskers extend to points within 1.5x IQR).

(**E**) The correlation between function scores (pegRNA-derived) and endogenous function scores is plotted for n = 141 variants passing the high-stringency threshold.

Figure S12

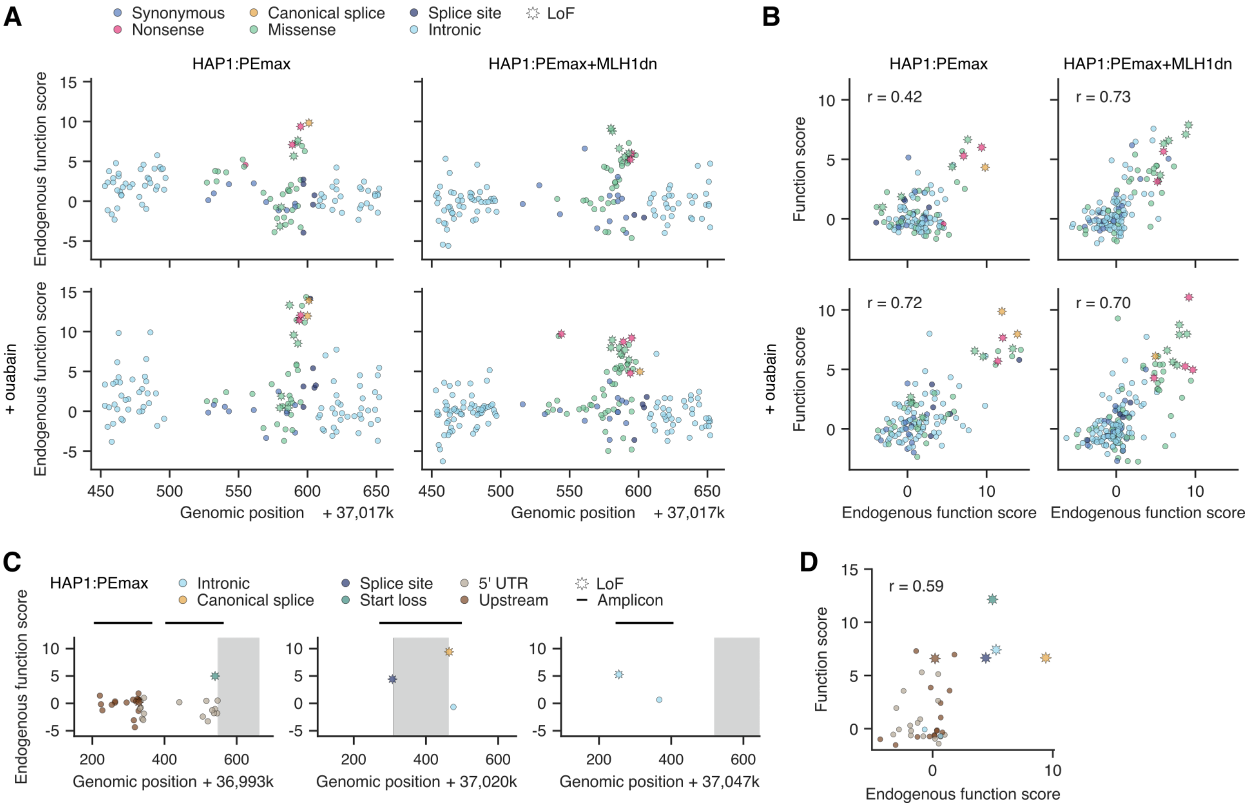

Figure S12. Amplicon sequencing of edited *MLH1* loci validates functional effects of variants installed via PE, related to Figures 3 and 4.

(**A**) Endogenous function scores of exon 10 variants are plotted by genomic position across conditions. Variants deemed LoF via pegRNA sequencing of all conditions are starred.

(**B**) Correlations between function scores and endogenous function scores are plotted for each experiment.

(**C**) Endogenous function scores are plotted by genomic position for each non-coding region validated. Endogenous function scores were derived by sequencing edited loci in HAP1:PEmax cells pre- and post-6TG selection.

(**D**) Correlation between function scores and endogenous function scores from HAP1:PEmax for regions in (**C**).

Figure S13

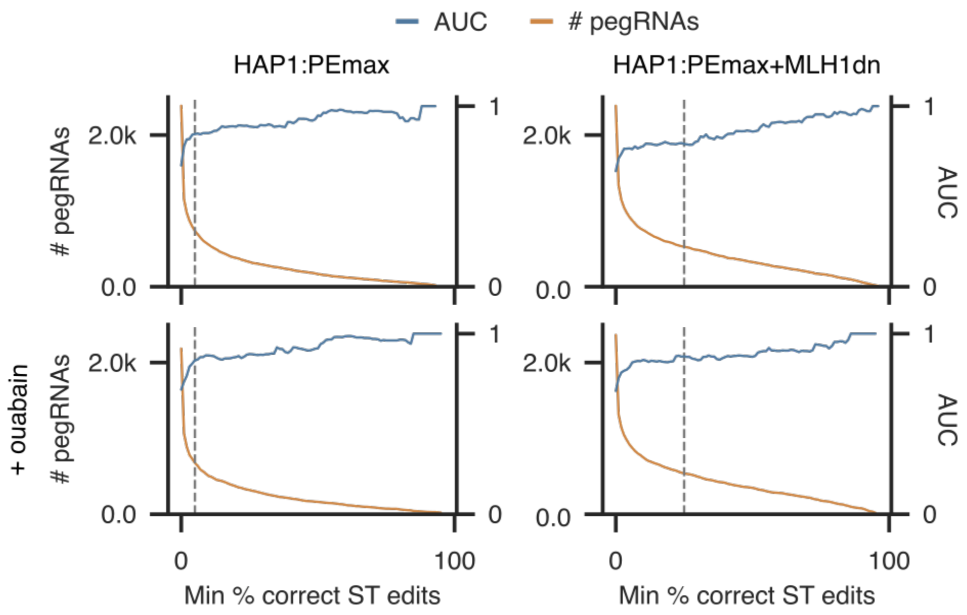

Figure S13. Accurate identification of LoF variants in the *MLH1* non-coding screen is facilitated by implementing ST editing thresholds, related to Figure 4. AUC measurements for distinguishing pLoF variants (blue line) are plotted as a function of ST editing threshold, shown for each experimental condition. The number of pegRNAs retained at each ST editing threshold is plotted in orange. For these experiments, pNeut variants were defined as intronic variants greater than 8 bp from exonic sequence and pLoF variants were defined as canonical splice site variants.

Figure S14

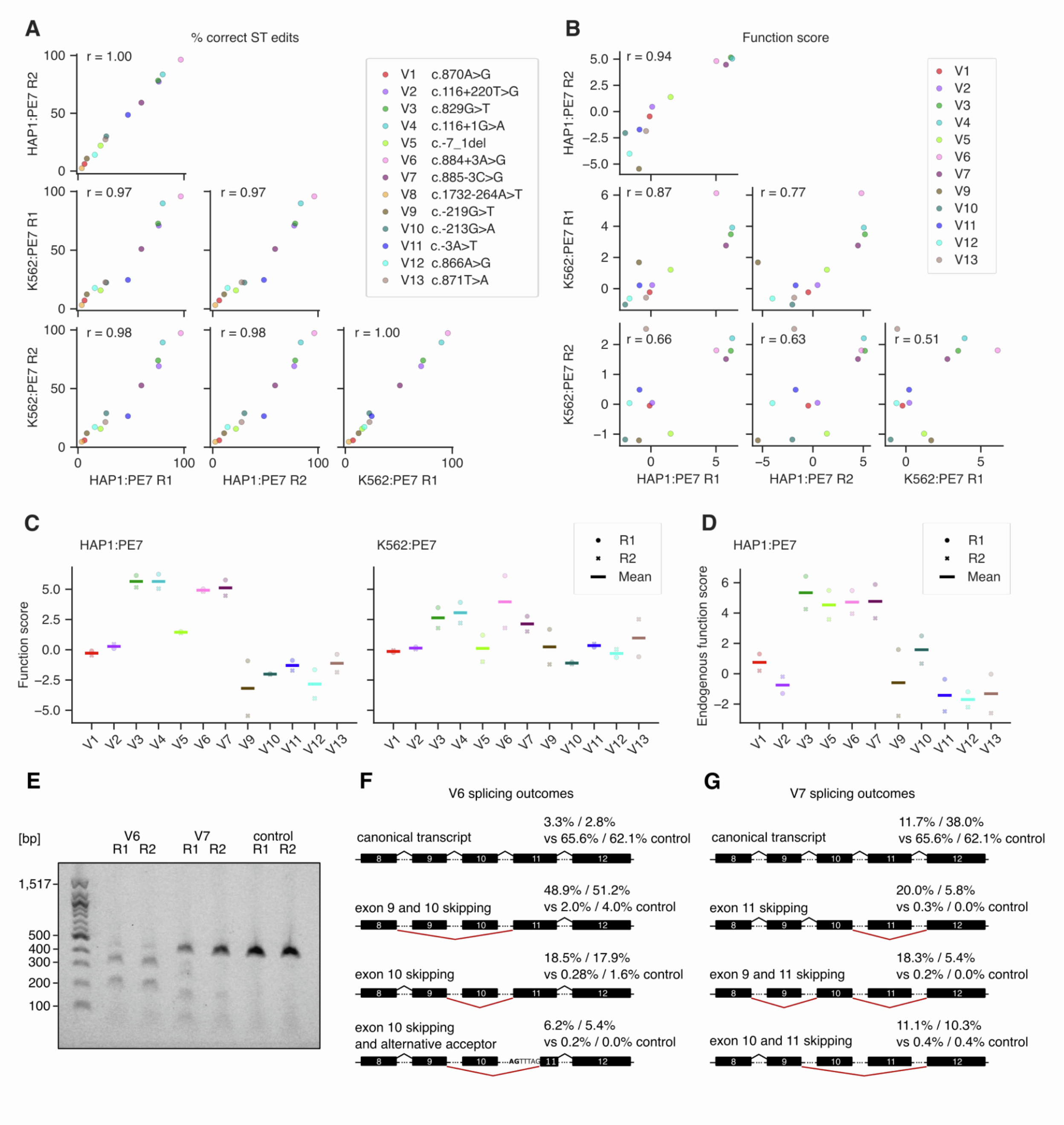

Figure S14. Experimental validation of *MLH1* variant effects, related to Figures 3 and 4.
(**A**) Correlations of correct ST-editing percentages on day 19 across replicates and cell lines for n = 13 variants re-tested in the *MLH1* minipool with newly designed pegRNAs. Pearson correlation coefficients (r) are shown.
(**B**) Correlations of function scores across replicates and cell lines are shown for n = 12 variants in the minipool with Pearson correlation coefficients (r) for each comparison. V8 was excluded from analysis due to low editing efficiency (less than 5% ST editing on D19).
(**C**) Final function scores were calculated by averaging function scores across HAP1:PE7 replicates (left) or K562:PE7 replicates (right), and by normalizing to the median of neutral controls (V1 and V2).
(**D**) Endogenous function scores are shown for n = 11 variants tested in HAP1:PE7 (excluding V8 due to insufficient editing activity and V4 due to lack of suitable primers).

(**E**-**G**) Potential splicing defects caused by V6 (c.884+3A>G) and V7 (c.885-3C>G) were investigated using RT-qPCR, gel electrophoresis and NGS. RT-qPCR products for V2 (a neutral control) V6, and V7 were generated using primers binding within exon 8 and across the exon 11-12 junction (**E**). NGS analysis of the resulting products for V6 (**F**) and V7 (**G**) reveals the loss of reads corresponding to canonical splicing and increased exon-skipping events. The three most abundant non-canonical isoforms detected are shown for each of V6 and V7, with the percentage of reads matching each product provided for each of n = 2 biological replicates compared to control (V2).

Supplemental references

1. Clement, K., Rees, H., Canver, M.C., Gehrke, J.M., Farouni, R., Hsu, J.Y., Cole, M.A., Liu, D.R., Joung, J.K., Bauer, D.E., et al. (2019). CRISPResso2 provides accurate and rapid genome editing sequence analysis. Nat. Biotechnol. *37*, 224–226.
2. Doench, J.G., Fusi, N., Sullender, M., Hegde, M., Vaimberg, E.W., Donovan, K.F., Smith, I., Tothova, Z., Wilen, C., Orchard, R., et al. (2016). Optimized sgRNA design to maximize activity and minimize off-target effects of CRISPR-Cas9. Nat. Biotechnol. *34*, 184–191.
3. Sanson, K.R., Hanna, R.E., Hegde, M., Donovan, K.F., Strand, C., Sullender, M.E., Vaimberg, E.W., Goodale, A., Root, D.E., Piccioni, F., et al. (2018). Optimized libraries for CRISPR-Cas9 genetic screens with multiple modalities. Nat. Commun. *9*, 1–15.
4. Mathis, N., Allam, A., Kissling, L., Marquart, K.F., Schmidheini, L., Solari, C., Balázs, Z., Krauthammer, M., and Schwank, G. (2023). Predicting prime editing efficiency and product purity by deep learning. Nat. Biotechnol. *41*, 1151–1159.
